# Impact of Chemical Corrosion on Mechanical Properties of Boroaluminosilicate Pharmaceutical Glasses

**DOI:** 10.3390/ma17133120

**Published:** 2024-06-25

**Authors:** Xinlin Ma, Jin Liu, Jun Zhang, Yucai Su, Kangfeng Yi, Yanfei Zhang, Linfeng Ding, Qiuju Zheng

**Affiliations:** 1 School of Materials Science and Engineering, Qilu University of Technology, Jinan 250353, China; 17861406968@163.com (X.M.); zhang-yanfei@hotmail.com (Y.Z.); 2 State Key Laboratory for Modification of Chemical Fibers and Polymer Materials, Engineering Research Center of Advanced Glass Manufacturing Technology, Ministry of Education, Donghua University, Shanghai 201620, China; linfeng.ding@dhu.edu.cn; 3 Shandong Pharmaceutical Glass Co., Ltd., Zibo 256100, China; zhangui@163.com (J.Z.); 15864953096@sina.cn (Y.S.); 1ykf@163.com (K.Y.)

**Keywords:** pharmaceutical glass, congruent dissolution, mechanical properties, nano-indentation

## Abstract

Boroaluminosilicate (BAS) glasses have excellent chemical durability and mechanical properties and are widely used in the pharmaceutical packaging industry. The corrosion behavior of boroaluminosilicate (BAS) glasses have been investigated for many years; however, the impact of chemical corrosion on mechanical properties of boroaluminosilicate glasses has not been well understood. In this work, the BAS glass samples were corroded in a 20 mM Glycine–NaOH buffer solution (pH = 10) at 80 °C for various durations. Within the corrosion durations, the corrosion of the glass is dominated by congruent dissolution. The results show that the elemental composition and structure of the glass surface are not altered significantly during the congruent dissolution, and the corrosion rate is mainly affected by the Si concentration in the solution. The structural change in the process of micro-crack decay is the main factor affecting the mechanical properties of the glass surface. Corrosion leads to the growth of micro-cracks and tip passivation, which causes the hardness and elastic modulus of the glass to first decrease and then increase. As corrosion proceeds, the microcracks are completely destroyed to form micropores, and the pore size and number increase with the corrosion process, resulting in the decrease in surface mechanical properties again. This work reveals the main influencing factors of congruent dissolution on mechanical properties and provides an important reference for the improvement of pharmaceutical glass strength.

## 1. Introduction

As the global outbreak of Corona Virus Disease (COVID-19) in 2019 [1,2], the demand for medicines and vaccines has increased dramatically. Therefore, more pressure has been put on the production and development of the pharmaceutical packaging materials industry [3]. Pharmaceutical packaging materials must be able to ensure the safety and effectiveness of drugs in storage and transportation [4,5]. The high chemical durability of borosilicate glasses could prevent medicines from being contaminated, and the high thermal stability enables them to bear processes such as high temperature sterilization and low temperature storage, and the excellent mechanical properties reduce the occurrence of damage during transportation and daily use [6,7,8,9,10].

The chemical durability of pharmaceutical glass is crucial to ensure the quality of drugs, and high chemical stability can effectively prevent the migration of harmful substances [11,12]. In the process of transportation and storage, glass will inevitably interact with external substances, and its excellent mechanical properties can ensure that it has sufficient resistance towards external forces such as friction and extrusion [13,14,15]. However, pharmaceutical glass will corrode during long-term contact with drugs, resulting in changes of mechanical properties [16,17]. Understanding the corrosion mechanism and the influence of corrosion on its mechanical properties is critical to improve the performance of the glass in practical application conditions.

The corrosion behaviors of glass depend on composition and corrosion conditions, thus it is difficult to find a uniform model that applies to all corrosion situations [18,19,20]. A large number of studies have shown that an alteration layer (AL) is often created on a glass surface in the long-term corrosion process, and the formation of the alteration layer will slow down the further corrosion process [21,22]. The microstructure of the alteration layer is extremely complex and very different from the original glass, such as “hydrated glass” or “mutual diffusion layer”, amorphous layer rich in silica, precipitate layer formed by secondary minerals and so on [23,24,25,26]. The complex microstructure of the alteration layer has a great influence on the mechanical properties of the glass surface [27,28]. Before the formation of the alteration layer, the congruent dissolution can often be observed, which involves microscopic reactions between the glass and the solution, such as hydrolysis, separation and transportation [29,30,31]. The congruent dissolution often occurs in the short-term corrosion stage, and it is only active in the corrosion front, i.e., the interface between the glass and solution. However, it does not change the chemical composition and microstructure of the glass surface significantly, so the study of corrosion behavior and mechanical properties in the congruent corrosion stage is often ignored. Establishing the correlation between mechanical properties and the congruent dissolution process is helpful to enhance the mechanical properties of pharmaceutical glasses.

Here, a commercial BAS pharmaceutical glass (The glass was bought from Shandong Pharmaceutical Glass Co., Ltd., Zibo, China) is chosen since it is often used as vaccine vials. All glass samples were put into 50 mL 20 mM Glycine–NaOH buffer solution (pH = 10) and corroded at 80 °C for 0 h to 384 h. After the corrosion process, the weight loss rate of the glass were measured, and the morphology of the glass surface were characterized by atomic force microscope (AFM). The chemical structure and element distribution at the corrosion interface were determined by Raman spectroscopy and time-of-flight secondary-ion mass spectrometer (TOF-SIMS), and it was confirmed that the corrosion process was the congruent dissolution stage without alteration layer formation. The mechanical properties of pharmaceutical BAS glass under different corrosion times were systematically studied by nanoindentation experiments. Based on the experimental results, the corrosion mechanism of BAS glass at the congruent dissolution stage and the mechanical properties evolution during corrosion are discussed.

## 2. Materials and Method

The commercial BAS pharmaceutical glasses exhibit superior chemical durability and mechanical properties. The chemical composition of the BAS glass is shown in Table 1. The BAS pharmaceutical glasses were cut into 10 mm × 10 mm × 5 mm, and the surface is fine-polished. Before the corrosion experiments, the samples were cleaned with distilled water, then washed with acetone, and finally dried in the oven for 6 h. For the static corrosion tests, we have followed the standard USP1660 proposed in United States Pharmacopoeia. The glass samples were put into 50 mL 20 mM Glycine–NaOH buffer solution with a pH of 10 and corroded at 80 °C for 0 h to 384 h. After the corrosion test, the sample is cleaned and dried for surface characterization.

The weight loss rate of the glass is calculated from the mass of the pristine glass and the corroded glass, and the glasses were weighed by the analytical balance (ME55/02, Mettler Toledo, Zurich, Switzerland, ±0.00001 g). The surface morphology of the BAS glass was characterized under the tapping mode of AFM (Multimode 8, Bruker, Billerica, MA, USA). The scanning range is 10 μm × 10 μm, with an accuracy of 1 nm for the *X*-axis and *Y*-axis, and 0.1 nm for the *Z*-axis. Raman spectroscopy (InVia Reflex, Renishaw, Gloucestershire UK) was used to analyze the chemical structure of the glass surface with an excitation wavelength of 532 nm, and it is in the range from 200 to 1400 cm^−1^ with a resolution of 2 cm^−1^.

The depth profile of each element was determined by time-of-flight secondary-ion mass spectrometer (TOF-SIMS 5-100, ION-TOF GmbH, Hamburg, Germany). The ion beam energy of the primary ion beam Bi^+^ is 30 keV, the incidence angle with the sample surface normal is 45°, and the scanning area is 50 μm × 50 μm. The ion beam energy of the sputtering ion beam O_2_^+^ is 2 kV, the incidence angle with the sample surface normal is 45°, and the sputtering area is 180 μm × 180 μm. The sputtering time can be converted into the sputtering depth after measuring the depth of the pit with a step profiler.

Nano-indentation behavior of the BAS glass before and after corrosion was studied by a nano-indentation instrument (Agilent G200, Keysight, Santa Rosa, CA, USA). Nano-Vision mode was applied to obtain the 3D shape of the indentation. The sharp cube-corner indenter (TC27120) was selected to obtain the indentation morphology. In this mode, a single load–unload cycle was first conducted with a maximum pressing depth of 500 nm. The indenter approaches the surface at the scan approach rate until the harmonic stiffness increases by surface stiffness. The load is increased to the scan load, and the 3D mapping begins; the scanning range is 3.5 μm × 3.5 μm. Then, the continuous stiffness mode (CSM) was applied to obtain the hardness and elastic modulus of the glass. To ensure the accuracy of the tests, the Berkovich tip (TB26082) was selected, and the applied load was set to be 200 mN. To ensure repeatability, all nanoindentation tests were independently repeated 15 times.

## 3. Results and Discussion

### 3.1. Weight-Loss Ratio

**Figure 1:**
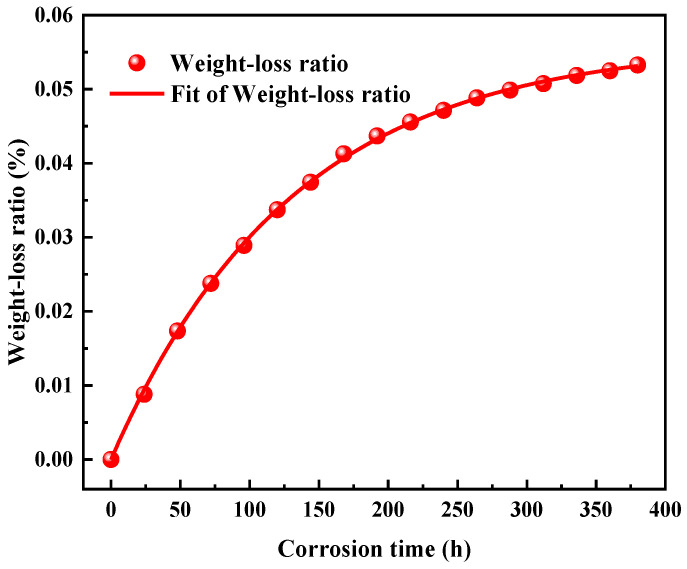
Weight loss ratio of the BAS glass samples as a function of time. An exponential function was used to fit the data and serve as a guideline to the eyes.

The weight loss rate is defined as mass loss divided by the initial mass of the glass, which can directly reflect the process of glass corrosion. The weight loss is mainly due to the dissolution of the glass network into the solution. The dissolution of the glass network usually occurs in alkaline solutions, where the silicate network is attacked by hydroxyl ions (OH^−^), and the surfaces of the glass exposed to the solution are rich in ≡Si-OH groups. The trend of the weight loss rate of the BAS glass as a function of time is shown in Figure 1. The weight loss rate of BAS glass increases the fastest in the early stage of corrosion, while the growth rate gradually decreases with the progress of corrosion. The whole corrosion process of glass is mainly controlled by the dissolution rate of the glass network, and the change in weight loss rate has a strong correlation with the concentration of Si dissolved into the solution [32]. As the corrosion proceeds, the concentration of Si in the solution increases, which lowers the dissolution rate of the glass network, thus the weight loss rate becomes slower in the late stage.

### 3.2. Surface Morphology and Roughness 

**Figure 2:**
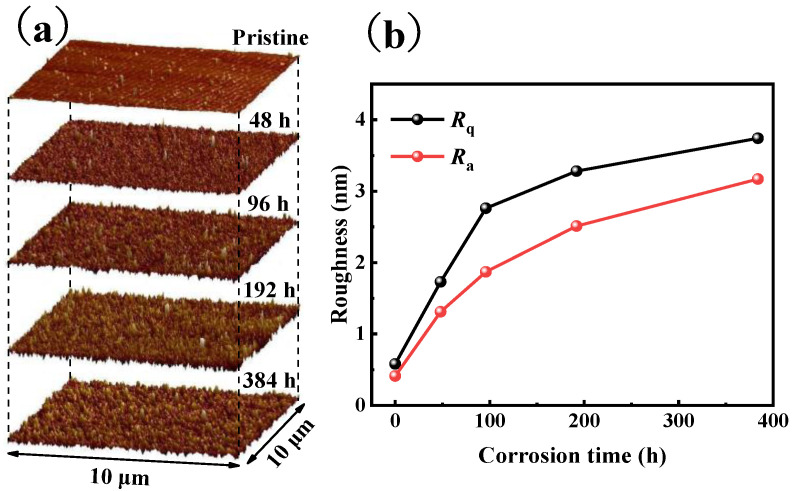
(**a**) AFM images of the BAS glasses under various corrosion conditions. (**b**) Surface roughness evolution of the BAS glasses under various corrosion conditions.

In order to obtain the changes in surface morphology and roughness with corrosion time, atomic force microscopy (AFM) was conducted on all the glass samples. The three-dimensional morphology of the samples before and after corrosion is shown in Figure 2a. The surface of the original glass sample is smooth and relatively flat except for a few scratches in the preparation process. As the corrosion progresses, the surface of the glass begins to appear like small, undulating peaks, evenly distributed and of similar height. The height and size of the surface protrusions increase with the increase in time, but the surface can still maintain a relatively flat state, and there is no obvious increase in local protrusions or a large area of deep grooves. The change in glass morphology during corrosion indicates that the network structure of glass has been altered during corrosion, and a large number of network forming groups are dissolved into solution.

The influence of corrosion time on glass surface roughness is shown in Figure 2b. The surface roughness parameters *R*_q_ (Arithmetic mean roughness) and *R*_a_ (RMS roughness) of the original sample were 0.582 nm and 0.410 nm, respectively. Compared with the original sample, the surface roughness parameters of the sample with corrosion time of 48 h, 96 h, 192 h, and 384 h were significantly increased. The roughness increases rapidly in the early stage of corrosion but slows down gradually with the progress of corrosion, which is similar to the change in weight loss rate (Figure 1). The variation in glass surface roughness before and after corrosion shows that its growth rate is controlled by the corrosion rate, and the corrosion rate gradually slows in the whole corrosion process.

### 3.3. Chemical Structure Change in Glass Surface

**Figure 3:**
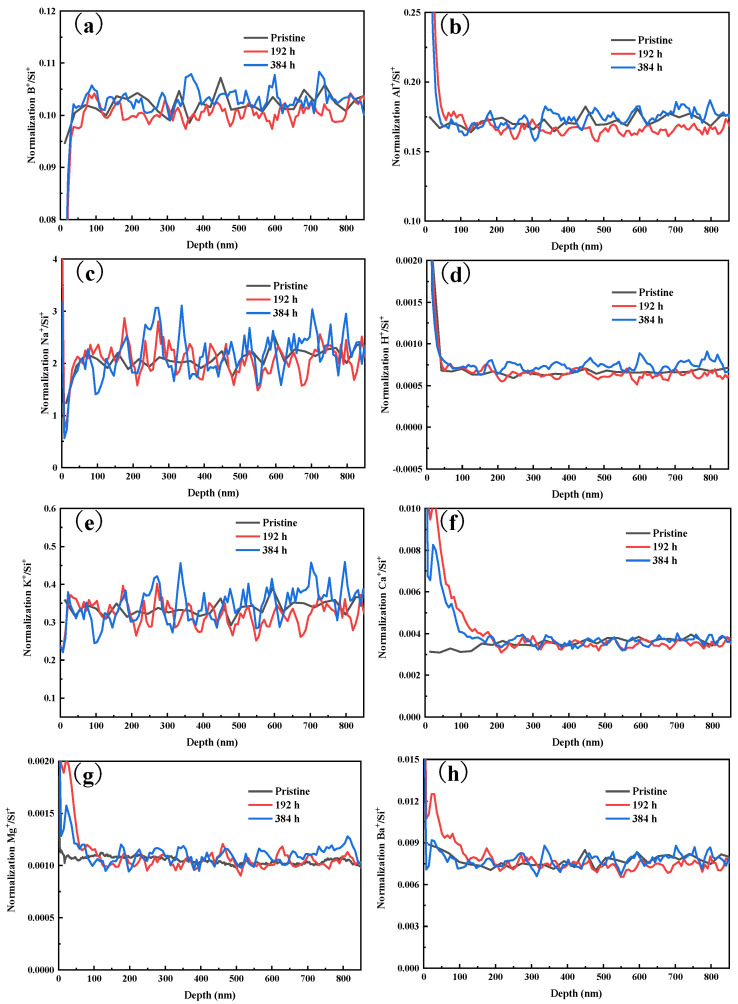
Depth profiles of four elements on the BAS glass surfaces under various corrosion conditions determined by TOF−SIMS: (**a**) B, (**b**) Al, (**c**) Na, (**d**) H, (**e**) K, (**f**) Ca, (**g**) Mg and (**h**) Ba, the intensity of each element is normalized by Si intensity.

In order to have a clearer understanding of the entire corrosion process, TOF−SIMS was used to detect the depth profiles of various elements on the glass surface with different corrosion durations, as shown in Figure 3. We can see that there is no significant difference in the depth profiles of B, Al, Na, H, K, Ca, Mg and Ba before and after corrosion, which is the result of the congruent dissolution of glass. The formation of an alteration layer on the glass surface is not observed. We can only observe subtle changes on the very surface (<50 nm) of the glass for certain elements. Moreover, the concentrations of each element as a function of depth become more fluctuated when the corrosion durations are longer, which corresponds well with the change in surface roughness (Figure 2). In the corrosion process, the dissolution reaction and the reprecipitation of amorphous silica take place on the glass surface simultaneously, which makes the dissolution–precipitation interface move forward [33,34]. The concentration of Si in solution are increased due to the dissolution of the glass network [35], which slows down the growth rate of glass weight loss (Figure 1).

**Figure 4:**
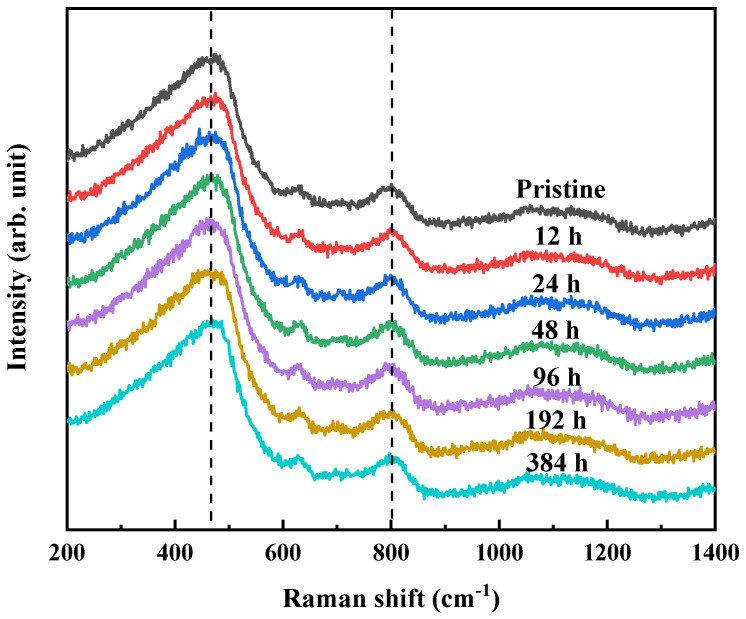
Raman spectra of glass surface under various corrosion durations.

To further explore the microstructure change on the glass surface, the Raman spectra of the BAS glass under various corrosion durations are shown in Figure 4. The Raman spectra of the BAS glasses can be divided into three main frequency regions: the low frequency region (250–750 cm^−1^), the intermediate frequency region (750–850 cm^−1^) and the high frequency region (850–1300 cm^−1^) [36]. In the low frequency region, there is an obvious wide peak near 480 cm^−1^, which is caused by the vibration of bridging oxygen and network formers (BO-T) [37]. In the intermediate frequency region, there is an obvious peak near 800 cm^−1^, which represents Si-O stretching involving oxygen motions in the Si-O-Si plane 38. The broadband in the higher frequency region usually refers to the vibration of non-bridging oxygen and network formers (NBO-T) [38]. By comparing the Raman spectra of BAS glass before and after corrosion, it can be seen that the intensity and position of each peak after corrosion do not change significantly, indicating that the surface structure of the glass does not change dramatically during the process of congruent dissolution.

### 3.4. Mechanical Properties of Glass 

**Figure 5:**
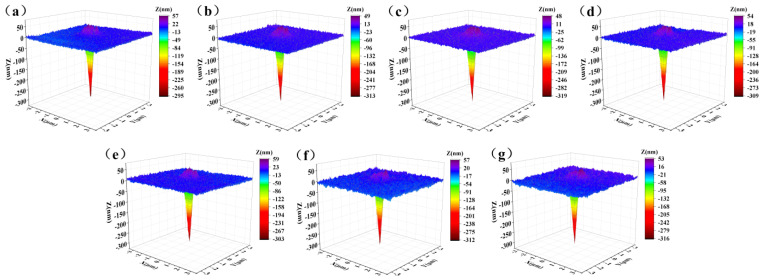
The three-dimensional (3D) images of indentation on different glass surfaces: (**a**) Pristine, (**b**) 12 h, (**c**) 24 h, (**d**) 48 h, (**e**) 96 h, (**f**) 192 h and (**g**) 384 h.

The 3D images of the indentation on various glass surfaces are obtained by nano-indentation tests. From Figure 5, it can be clearly seen that after unloading, the indenter is divided into the remaining indentation part under the glass surface and the pile-up part at the edge of the indentation above the glass surface. The indentation deformation behavior of glass surface under pressure mainly includes elastic deformation (it will recover immediately after unloading), densification (forming a relatively compact packing structure) and shear flow (maintaining volume conservation and causing pile-up around the indentation edge) [39,40,41].

**Figure 6:**
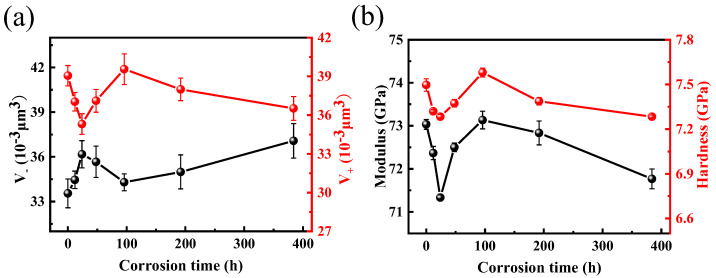
(**a**) The pile-up volume (*V*^+^) and residual indentation volume (*V*^−^) of the indentation as a function of corrosion time. (**b**) Hardness and elastic modulus of the BAS glass with various corrosion durations.

The pile-up part is generated by shear flow during the indenting process to maintain the conservation of volume. The maximum pile-up height as a function of corrosion time decreases first and then increases, and finally, it decreases again. The residual indentation depth shows an opposite trend to the maximum pile-up height. In order to evaluate the volume changes of each part of indentation deformation during the entire corrosion process more accurately, a newly developed three-dimensional surface analysis method [42] was used to quantify the pile-up volume (*V*^+^) and residual indentation volume (*V*^−^) of the indentation, and the results are shown in Figure 6a.

It can be seen from Figure 6a that the changes in the pile-up volume (*V*^+^) as a function of corrosion time follows three stages: (1) it decreases sharply with short corrosion times, (2) it increases greatly in the medium corrosion times, and (3) it decreases gradually with long corrosion times. The trend of residual indentation volume (*V*^−^) shows an opposite variation. If elastic deformation is the main factor causing the volume change, volume conservation can be maintained with less shear flow when the large elastic deformation is generated. In this case, the pile-up volume (*V*^+^) will be smaller, while the recovery amount of elastic deformation after unloading is larger, resulting in a smaller indentation residual volume. Then, the pile-up volume *V*^+^ will be positively correlated with the residual indentation volume (*V*^−^), which is obviously inconsistent with the actual situation. Therefore, the elastic deformation is not the main factor causing the volume change. Shear flow and densification together control the volume change of each part of the indentation. 

The TOF-SIMS (Figure 3) and Raman spectra (Figure 4) data show that the structure and element distribution on the glass surface do not change significantly during the whole corrosion process, thus the change in the indentation volume is not caused by the change in the chemical composition on the glass surface. There are various defects on the glass surface and inside of the bulk glass when glass is formed, and the fine polishing process could cause micro-cracks and other defects on the glass surface also [43,44,45]. Therefore, the actual strength of the glass is several orders of magnitude lower than the theoretical strength, and the strength of the glass after grinding and polishing will be reduced to some extent [46,47,48].

The micro-crack passivation mechanism [49] can well explain the phenomenon of the indentation volume change. At the initial stage of corrosion (0−24 h), the corrosion liquid penetrated in the micro-cracks makes them grow, and when the indenter is pressed into the glass, it is easier to produce stress concentration at the crack tip. Due to the stress concentration inside the crack, the local glass will be densified higher under the action of stress, and the crack will continue to expand under the action of stress, resulting in the local high-densification area increasing. At this stage, the pile-up volume (*V*^+^) generated by shear flow to maintain volume conservation decreases, the local high densification region generated near the micro-crack region after unloading can continue to be maintained, and the residual indentation volume *V−*^−^ after indentation unloading increases. In the middle stage of corrosion (24−96 h), as the corrosion proceeds, the tip of the microcrack is passivated by corrosion, and the further expansion of the microcrack is also hindered when the indenter is pressed into the glass. After the expansion of the microcrack is hindered, the local high densification area generated by the stress concentration is reduced. The change in the volume of each part of the indentation after unloading is opposite to the change in the crack growth stage at the beginning of corrosion. In the late stage of corrosion (96−384 h), due to long-time corrosion, the original micro-cracks are destroyed, and a porous structure is formed. The pores may be connected with each other, making the glass surface loose and porous. When the indenter is pressed into the glass, the pores collapse under pressure, and the whole structure becomes denser. The pore diameter and number of pores increases with the corrosion time, and the pile-up volume (*V*^+^) decreases. The collapsed pores cannot be recovered after unloading, and the residual indentation volume (*V*−) increases with the corrosion time.

The hardness (*H*) and elastic modulus (*E*) of the glasses corroded at different times are shown in Figure 6b, and their variation trends correlate with the change in the pile-up volume (*V*^+^) well. We further verify the influence of micro-cracks on the surface properties of the glass during corrosion. In the growth stage of the micro crack tip, the micro cracks expand along the direction of the micro crack propagation under the continuous accumulation of stress, resulting in the decrease in the hardness and elastic modulus of the glass. After the microcrack tip is destroyed, the further propagation of the microcrack will be hindered when the indenter is pressed into the glass, and the hardness and elastic modulus of the glass will be restored. As corrosion becomes more intense, the original micro-cracks are destroyed by long-time corrosion, and the glass surface becomes loose and porous. The pore size and number of pores continue to increase with the corrosion process, and the hardness and elastic modulus of the glass decrease with the increase in corrosion time.

## 4. Conclusions

The impact of chemical corrosion on mechanical properties of the BAS pharmaceutical glasses have been investigated. The results show that the structure and element distribution of the glass surface are not changed significantly during the congruent dissolution process, and the corrosion rate is mainly affected by the concentration of Si in the solution. With the progress of corrosion, the roughness of the glass surface increases obviously. The micro-crack passivation mechanism is used to explain the change in the mechanical properties as a function of corrosion time. Corrosion leads to the growth of micro-cracks and the passivation of the tip, which makes hardness and elastic modulus of the glasses decrease first and then increase. Finally, the microcracks are completely destroyed and form micropores, resulting in the decrease in mechanical properties again. This work is helpful for the improvement of the mechanical properties of pharmaceutical glasses.

## Figures and Tables

**Table 1:** Chemical composition of the pharmaceutical BAS glass.

Composition	SiO_2_	B_2_O_3_	Al_2_O_3_	Na_2_O + K_2_O	MgO + CaO + BaO + SrO
Content (wt%)	75	≥8	2–7	4–8	5

## Data Availability

The data presented in this study are available on request from the corresponding author. The data are not publicly available due to [legal reasons].
